# Affective temperament in inflammatory bowel diseases: Another brick in the wall of differentiation

**DOI:** 10.1371/journal.pone.0205606

**Published:** 2018-11-01

**Authors:** Maciej Bieliński, Natalia Lesiewska, Joanna Bielińska, Ariel Liebert, Artur Mieczkowski, Paulina Sopońska-Brzoszczyk, Bartosz Brzoszczyk, Maria Kłopocka, Alina Borkowska

**Affiliations:** 1 Chair and Department of Clinical Neuropsychology, Nicolaus Copernicus University, Toruń, Poland, and Collegium Medicum, Bydgoszcz, Poland; 2 The Institute of Gastroenterologic Nursing, Nicolaus Copernicus University, Toruń, Poland, and Collegium Medicum, Bydgoszcz, Poland; 3 Department of Obstetrics and Gynecology, Nicolaus Copernicus University, Toruń, Poland, and Collegium Medicum, Bydgoszcz, Poland; 4 Department of Urology, Dr Jan Biziel University Hospital No.2, Bydgoszcz, Poland; Universita degli Studi Europea di Roma, ITALY

## Abstract

Psychiatric disorders are significantly common complications among patients suffering from inflammatory bowel diseases (IBD). Affective temperament is a concept of core personality traits, which can decribe the vulnerability to mood disorders, therefore its evaluation might convey useful information about patients' mental status in autoimmune disorders. The aim of the study was to evaluate the affective temperament in patients with Crohn's disease (CD) and ulcerative colitis (UC) as characteristic features of these diseases, but also in the clinical course and the severity of anxiety and depression.Due to our knowledge this is the first study of this kind. The study enrolled 130 patients with IBD, including 68 with CD and 62 with UC. We used TEMPS-A to evaluate affective temperament and HADS scales to assess the intensity of depressive and anxiety symptoms. Harvey Bradshaw scale, Crohn’s Disease Activity Index (CDAI) and Mayo Score were used to evaluate clinical severity of the diseases. We observed significantly higher prevalence of depressive, cyclothymic and anxiety temperaments in CD patients compared to the control group. Harvey Bradshaw scale, CDAI and Mayo Self Report showed statistically significant outcomes, including significant positive correlations with depressive, cyclothymic and anxiety subscales of TEMPS-A, and negative correlation with the hyperthymic temperament in CD subjects. Our findings indicate significant differences between CD and UC due to temperament traits, and suggest distinct pathogenesis of mood disorders in IBD.

## Introduction

Inflammatory Bowel Disease (IBD), such us Crohn's Disease (CD) and Ulcerative Collitis (UC) are chronic and relapsing gastrointestinal disorders with crucial implications. This issue requires greater attention provided that the number of patients with IBD is still significantly increasing, especially in the western industrialized countries. The occurence rate of IBD in adult Europeans can reach even 140 per 100,000 inhabitants [1].

IBD may lead to several considerable complications associated not only with gastrointestinal manifestation, but can also affect other relevant systems [2–6]. This contributes to poor quality of life (QoL) in IBD patients. They present worries and concerns regarding complications, stigmatization or intimacy, as well as menagement plans or effective treatment interventions [7]. According to many studies, impaired QoL depends on the disease activity [8,9].

Epidemiological studies have established that anxiety and depression are the most prevalent psychiatric disorders among IBD patients [10–13]. Also patients suffering from other immune-mediated inflammatory diseases, such as multiple sclerosis or rheumatoid arthitis, are at the increased risk of psychiatric comorbidity [14,15]. The incidence of mood disorders in IBD is not well understood, although the risk of falling for depression or anxiety gets higher, as QoL decreases [15]. It has been noted, that long-term stress may change the course of the disease resulting in higher risk of exacerbations [16]. Therefore, in the case of enhanced symptoms during the course of IBD, it is crucial to take into account patient's mental health and stress levels [17].

Hagop Akiskal has developed the novel concept of affective temperament; it comprises inherited personality traits which may become the extreme manifestations of affective disorders. Affective temperament might be considered as a phenotype which derives from genetic and biological bases. It remains rather stable over time, however dysregulations of the temperament may predispose to the development of affective disorders, like depression or bipolar disease [18–20,21]. Hence, affective temperament may be considered as a factor predisposing to psychiatry diseases, such as depression or anxiety.

Temperament Evaluation of Memphis, Pisa, Paris and San Diego Autoquestionnaire (TEMPS-A) is a self-report instrument used in order to assess five affective temperaments in subjects: hyperthymic, depressive, irritable, cyclothymic and anxious [22]. Recent studies show numerous correllations between greater scores of temperaments which show liability to mood disorders and the prevalence of depressive or anxiety symptoms [23]. TEMPS-A has been proved useful in the temperament evaluation in context of both depressive and anxiety symptoms in autoimmune disorders [24–26]. However, currently there is no study describing temperament in patients suffering from IBD. Thus, the aim of this study was to evaluate the prevalence of five traits of affective temperament, by means of TEMPS-A, in IBD patients in relation to the intensity of depression and anxiety and clinical manifestation of the diseases in both UC and CD patients. We hypothesized that patients with IBD show specific affective temperament profile, which may be associated with clinical manifestation of the diseases, including neuropsychiatric symptoms e.g. depression and anxiety. To our knowledge, this is the first study laying the emphasis on the affective temperament in IBD, and its relation to the intensity of clinical factors affecting the course of both CD and UC.

## Materials and methods

### Participants

The study involved 130 patients; 68 with CD (30 women and 38 men) and 62 with UC (31 women and 31 men). They were of Polish nationality and white ethnicity. The mean age of participants was 28,5 years (range, 22–36,5 years) for CD group and 31 years (range, 21–50 years) for UC group. The patients were treated outpatient at the Clinic of Intestinal Diseases and, according to the consent of the bioethical commission, they were recruited on the basis of the proposal of a gastroenterologist. During the observation, the diagnosis has not been changed. Demographic factors are shown in Table 1.

**Table 1:** Basic demographic and clinical parameters in subgroups of patients with CD and CU.

		CD (n = 68)	UC (n = 62)	p	Cohen’s d
**Age (y)**		28.5 (22.0–36.5)	31.0 (21.0–50.0)	0.41	0.30
**Gender**		♀ 30 (44%) ♂ 38 (56%)	♀ 31 (50%) ♂ 31 (50%)	0.63	-
**Duration of illness (y)**		5.0 (3.0–8.0)	6.0 (2.0–8.0)	0.74	0.007
**Biometrics**	**BMI (kg/m^2^)**	22.1 (19.2–24.8)	22.5 (20.2–26.3)	0.19	0.36
	**Waist (cm)**	81.0 (76.0–89.0)	83.0 (76.0–90.0)	0.55	0.27
**Education**	**Basic (n,%)**	4 (6%)	4 (6.5%)	0.82	-
	**Vocational (n,%)**	11 (16%)	8 (13%)		
	**Secondary (n,%)**	33 (48.5%)	31 (50%)		
	**Higher (n,%)**	20 (29.5%)	19 (30.5%)		
**Physical Activity**	**None (n,%)**	25 (37%)	24 (39%)	0.60	-
	**< 1/week**	8 (12%)	14 (22.5%)		
	**2-3/week**	31 (45.5%)	16 (26%)		
	**>3/week**	4 (5.5%)	8 (12.5%)		
**Cigarette smoking (n, %)**		11 (16%)	14 (23%)	0.81	-

Values are expressed as the median (25–75%) or as number of patients (n). Significance of differences between groups was determined by Mann–Whitney U Test. BMI: Body Mass Index.

The inclusion criteria were the diagnosis of IBD and the signing of the Informed Consent Form for participation in the study. The the diagnosis of IBD was determined based on diagnostic criteria, including clinical presentation, laboratory, endoscopic and imaginary studies, as well as histological results.

In order to determine differences in the expression of temperament dimensions, the control group of healthy people was selected in terms of sex and age for the study groups.

Participants with a severe somatic or psychiatric disorder per *Diagnostic and Statistical Manual of Mental Disorders*, 4^th^ edition, or with diagnosed any neurological abnormality, addiction to illicit drugs or alcohol were excluded from the study.

### Ethical statement

Permission for the study was obtained from the Bioethical Commission of the Nicolaus Copernicus University, Collegium Medicum in Bydgoszcz. The subjects demonstrated their willingness to participate in the study by signing the Informed Consent Form. All the patients were informed about the goals and processes of the study, potential risks, anonymity of the data and the possibility to cancel their participation at any moment.

### Assessments

The procedure of examination shown below was conducted once and consisted of clinical, biochemical and psychological assessment.

### Clinical assessment

Clinical evaluation was based on physical examination and medical history as well as filling out a questionnaire concerning the course of the disease and individual factors. We evaluated demographic factors (age, sex, education, place of residence etc.), parameters related to illness: disease type, duration, other diseases, risk factors of IBD or exacerbations, anthropometric parameters etc.

### Assessment of disease activity

For the assessment of clinical activity and course of the disease, we utilized Crohn's Disease Activity Index (CDAI) and Harvey-Bradshaw Index for CD. For UC activity evaluation we used Mayo Classification.

#### Crohn’s Disease Activity Index

CDAI is a standard questionnaire used in research studies in order to assess disease activity. Calculation of CDAI involves 8 items such as hematocrit, physical examination outcomes, and 1-week diary data of the number of liquid stools, the intensity of abdominal pain and general well-being. Every parameter is evaluated in numeric scale and increasing number of points is associated with disease exacerbation. Another item involves the occurrence of concurrent complications such as arthritis, uveitis, erythema nodosum, fistulas or abscesses. Moreover CDAI includes the usage of anti-diarrhea drugs, and the presence of abdominal mass which is evaluate during physical examination. The disease activity is calculated building on the total amount of obtained points [27].

#### Harvey-Bradshaw Index

Harvey-Bradshaw Index is simplified alternative to the CDAI, designed for easier data collection and calculations in evaluating CD’s activity. In comparison to CDAI, this scale does not require a 7-day data collection from the patient and is based on the assessment from the 1-day observations. Evaluated items consist of general well-being, the intensity of abdominal pain, the daily number of liquid stools, the occurrence of abdominal mass and concurrent complications associated with UC. The disease activity is determined based on overall obtained points. Patients with score < 5 are likely to be in remission; scores over 5 points suggest exacerbation [28]. Studies show positive correlations between both CDAI and Harvey-Bradshaw index in CD activity assessment. [29–31].

#### The Mayo Score

The Mayo Score is used to assess the activity of UC. The scale shows patient’s symptoms (such as abdominal pain, rectal bleeding, the number of liquid stools), the appearance of the moid mucosa in endoscopic studies and other findings during physical examination. The sum of all obtained scores reflects disease activity [32].

### Psychological assessment

We used Temperament Evaluation of Memphis, Pisa, Paris and San Diego Autoquestionnaire (TEMPS-A) to assess affective temperament in our group of patients. The anxiety and depressive symptoms intensity were evaluated using the HADS scale (Hospital Anxiety and Depression Scale) with the cut-off score as over 8.

#### Temperament Evaluation of Memphis, Pisa, Paris and San Diego Autoquestionnaire (TEMPS-A)

TEMPS-A measures affective temperament traits, represented by five dimensions: depressive, cyclothymic, anxious, irritable and hyperthymic. The tool consists of 110 items; version for males contains 109 questions. Questions regarding each dimension require simple “yes” (score 1) or “no” (score 0) answers, and are grouped together in following manner:

1. questions 1 to 21 (21 points) relate to depressive temperament;
2. questions 22 to 42 (21 points) relate to cyclothymic temperament;
3. questions 43 to 63 (21 points) relate to hyperthymic temperament;
4. questions 64 to 84 (21 points, 20 points in the version for men) relate to irritable temperament;
5. questions 85 to 110 (26 points) relate to anxious temperament.

Points scored for each temperament are summed up and then divided by the number of questions pertained to each dimension. Based on that, the severity of each temperament is measured [18,33]. In our study, the Polish version of TEMPS-A was utilized [34].

#### Hospital Anxiety and Depression Scale (HADS)

HADS is a simple tool for the assessment of anxiety and depression in patients who suffer from somatic diseases [35]. The auto-questionnaire contains two parts: seven questions to evaluae the severity of depressive symptoms, and another seven questions applying to anxiety symptoms. Results for anxiety and depression are summed up separately, albeit the questions of each part are interspersed. We used following cut-off points established for Polish population: 0–7 –no disorders; 8–10 –mild intensity; 11–21 –severe intensity [36].

### Statistical analysis

STATISTICA 12.5 was used for statistical analyses; it was conducted for two groups. Using the Shapiro-Wilk test, it was found that the test group does not meet the normal distribution criteria. To assess the significance of the differences between groups the Mann-Whitney U test was used. Effect size was expressed using Cohen's d. The R-Spearman test was used to determine correlations between non-parametrical variables. Differences and correlations were considered significant for p <0.05.

## Results

The initial analysis of affective temperament showed that patients with CD have significantly more depressive (p = 0.018, d-Cohen = 0.51), cyclothymic (p = 0.026, d-Cohen = 0.32) and irritable (p = 0.029, d-Cohen = 0.27) dimensions in relation to patients with UC (Table 2). Similar analyzes were carried out regarding the subgroup results of patients with CD and UC to the results of the control group matched according to gender, age and level of education to the patients groups (Table 3). The results indicated higher prevalence of depressive (p = 0.0004, d-Cohen = 0.43), cyclothymic ((p = 0.0001, d-Cohen = 0.27) and anxiety (p = 0.001, d-Cohen = 0.52) temperaments among CD patients compared to the control group. In contrast, patients with UC were only characterized by a significantly lower scores of irritability in TEMPS-A.

**Table 2:** HADS and TEMPS-A results in subgroups of patients with CD and UC.

	CD (n = 68)	UC (n = 62)	p	Cohen’s d
HADS_A	7.0 (5.0–9.0)	6.0 (2.0–7.0)	0.69	0.09
HADS_D	4.0 (2.0–7.0)	4.0 (2.0–7.0)	0.90	0.12
TEMPS_depressive,	0.38 (0.28–0.48)	0.33 (0.19–0.43)	**0.018**	0.51
TEMPS_cyclothymic	0.43 (0.26–0.57)	0.28 (0.14–0.57)	**0.026**	0.32
TEMPS_hyperthymic	0.52 (0.33–0.57)	0.52 (0.38–0.67)	0.47	0.09
TEMPS_irritable	0.22 (0.1–0.35)	0.15 (0.09–0.23)	**0.029**	0.27
TEMPS_anxious	0.35 (0.19–0.58)	0.27 (0.15–0.44)	0.13	0.25

Values are expressed as the median (25–75%) or as number of patients (n). Significance of differences between subgroups was determined by Mann–Whitney U Test.

**Table 3:** TEMPS-A results in subgroups of patients with CD and UC and in control group.

	CD (n = 68)	Control (n = 132)	P	UC (n = 62)	Control (n = 132)	p
**Age (y)**	28.5 (22.0–37.0)	25.0 (24.0–30.0)	0.47	31.0 (20.0–50.0)	25.0 (24.0–30.0)	0.31
**Gender (F/M, n, %)**	♀ 30 (44%) ♂ 38 (56%)	♀ 73 (55%) ♂ 59 (45%)	0.11	♀ 31 (50%) ♂ 31 (50%)	♀ 73 (55%) ♂ 59 (45%)	0.39
TEMPS_depressive,	0.38 (0.28–0.48)	0.28 (0.19–0.43)	**0.0004**	0.33 (0.19–0.43)	0.28 (0.19–0.43)	0.51
TEMPS_cyclothymic	0.43 (0.26–0.57)	0.26 (0.14–0.45)	**0.0001**	0.28 (0.14–0.57)	0.26 (0.14–0.45)	0.34
TEMPS_hyperthymic	0.52 (0.33–0.57)	0.52 (0.33–0.67)	0.27	0.52 (0.38–0.67)	0.52 (0.33–0.67)	0.57
TEMPS_irritable	0.22 (0.1–0.35)	0.19 (0.09–0.30)	0.55	0.15 (0.09–0.23)	0.19 (0.09–0.30)	**0.039**
TEMPS_anxious	0.35 (0.19–0.58)	0.23 (0.12–0.38)	**0.001**	0.27 (0.15–0.44)	0.23 (0.12–0.38)	0.10

Values are expressed as the median (25–75%) or as number of patients (n). Significance of differences between subgroups and controls was determined by Mann–Whitney U Test.

The study of the correlation of the TEMPS-A scale with the total score of the CDAI clinical scale did not show significant results. However, a similar analysis with the total result of the Harvey Bradshaw scale, the higher the score expresses the greater the activity of the disease, showed significant positive correlations with depressive (p = 0.025), cyclothymic (p = 0.049) and anxiety (p = 0.007) affective temperaments, as well as negative ones with the hyperthymic dimension (Table 4). The number of surgical procedures was not related to the TEMPS-A subscales for both IBD groups.

**Table 4:** TEMPS-A correlation with clinical factors in CD.

CD	No of surgical inteventions		CDAI		Harvey-Bradshaw Scale	
	R	p	R	p	R	p
TEMPS_depressive,	0.105	0.39	0.218	0.07	0.27	**0.025**
TEMPS_cyclothymic	0.052	0.68	0.064	0.62	0.245	**0.043**
TEMPS_hyperthymic	0.125	0.33	-0.085	0.51	-0.239	**0.049**
TEMPS_irritable	0.11	0.37	-0.127	0.29	0.071	0.57
TEMPS_anxious	0.179	0.14	0.125	0.33	0.322	**0.007**

R—Spearman correlations

In the next step, the correlations between TEMPS-A and the clinical self-assessment parameters of CDAI and Harvey Bradshaw indices were analyzed (Table 5). Significant correlation of TEMPS-A only with the number of stools has been found. The higher scores on depressive (p = 0.003 for CDAI and p = 0.015 for HBS) and anxiety (p = 0.031 for CDAI and p = 0.012 for HBS) dimensions were associated with a higher number of stools, while the higher scores on hyperthymic temperament were associated with a significantly smaller number of stools (p = 0.024 for CDAI and p = 0.008 for HBS). Similar analysis was then carried out for the results of patients with UC and Mayo Clinical scale, divided into a medical evaluation and self-assessment (Table 6). Significant correlations were associated only with self-assessment parameters. More pronounced subjective symptoms of the disease were significantly positively correlated with cyclothymic (p = 0.038) and anxiety (0.031) dimensions of TEMPS-A. The positive correlation of the depressive scale remained in the trend. The negative significant correlation was related to the hyperthymic temperament (p = 0.034).

**Table 5:** TEMPS-A correlation with the dimensions of subjective symptoms of scales assessing the clinical course of the Crohn Disease.

CD	CDAI			Harvey-Bradshaw Scale	
	No of soft stools	Abdominal pain	General well-being	No of soft stools	General well-being
TEMPS_depressive,	**0.352** **p = 0.003**	0.054	0.163	**0.292** **p = 0.015**	0.222
TEMPS_cyclothymic	0.232	-0.054	-0.018	0.230	0.187
TEMPS_hyperthymic	**-0.273** **p = 0.024**	-0.150	0.077	**-0.318** **p = 0.008**	-0.081
TEMPS_irritable	0.099	-0.066	-0.137	0.061	-0.004
TEMPS_anxious	**0.26*1*** **p = 0.031**	0.107	0.115	**0.303** **p = 0.012**	0.226

**Table 6:** TEMPS-A correlation with clinical factors in Ulcerative Colitis. R- Spearman correlations.

UC	No of surgical inteventions		Mayo-Physicians assessment		Mayo-Self assessment	
	R	p	R	p	R	p
**TEMPS_depressive,**	-0.056	0.66	-0.036	0.78	0.226	0.077
**TEMPS_cyclothymic**	0.073	0.57	0.183	0.15	0.264	**0.038**
**TEMPS_hyperthymic**	-0.09	0.48	-0.113	0.38	-0.27	**0.034**
**TEMPS_irritable**	-0.128	0.32	0.128	0.32	0.191	0.13
**TEMPS_anxious**	-0.186	0.14	0.034	0.79	0.273	**0.031**

R—Spearman correlations

Table 7 presents the results of the correlation between the severity of anxiety and depression symptoms measured with HADS and the dimensions of affective temperament. Hyperthymic temperament in both study subgroups was associated with lower symptoms of anxiety and depression, however, significant correlations were related only to the severity of anxiety. The other dimensions of affective temperament exhibited statistically significant positive correlations with the severity of anxiety and depression, except for the irritable temperament, which did not reach significance in patients with CD.

**Table 7:** TEMPS-A correlation with anxiety and depression (HADS).

	CD (n = 68)				UC (n = 62)			
	HADS_A		HADS_D		HADS_A		HADS_D	
	R	p	R	p	R	p	R	p
**TEMPS_depressive**	0.43	**0.002**	0.31	**0.01**	0.41	**0.0009**	0.53	**<0.0001**
**TEMPS_cyclothymic**	0.47	**<0.0001**	0.33	**0.006**	0.49	**<0.0001**	0.56	**<0.0001**
**TEMPS_hyperthymic**	-0.16	0.19	-0.40	**0.0007**	-0.10	0.44	-0.45	**0.0002**
**TEMPS_irritable**	0.41	**<0.0001**	0.14	0.25	0.34	**0.007**	0.33	**0.009**
**TEMPS_anxious**	0.58	**<0.0001**	0.37	**0.002**	0.53	**<0.0001**	0.58	**<0.0001**

R—Spearman correlations

An analysis of covariance was also carried out for the effects assessment of gender, age and education on HADS results and parameters related to disease activity. In the CD group of patients, gender was the only parameter which affected the model with statistical significance (F = 3.8, p = 0.008). However, among patients with CU model was significantly affected by gender (F = 12.9, p = 0.004) and education (F = 3.6, p = 0.008).

## Discussion

Temperament displays the relatively stable biological and genetic basis of personality which can putatively affect the risk of psychiatric comorbidities [22]. Mood disorders are very prevalent among IBD patients and by their intensity create two-dimensional dependencies with the clinical course. Hence TEMPS-A, as a simple tool, can be very useful in assessing patient's morbidity to psychiatric disorders [37]. To our knowledge, this is the first study scrutinizing affective temperament in patients suffering from CD and UC.

As was shown in Table 2, CD patients scores were significantly higher in depressive, cyclothymic and irritable temperaments in comparison to UC patients. This may imply that patients suffering from CD may be more vulnerable to mood disorders, such as depression and anxiety, than UC patients. However, we have not observed any differences in HADS scales between those diseases. Researches describing mood disorders in IBD, usually assess both UC and CD collectively (as IBD) instead of evaluating them individually [38–40]. Our findings suggest that the origin of psychiatric symptoms in CD and UC may ensue from different mechanisms. More research is needed in this regard to invent adjusted strategies aiming to treat psychiatric comorbidities in particular group of IBD patients.

Subsequently, we compared TEMPS-A scales in CD and UC patients versus control groups. The results indicated that more strongly expressed depressive, cyclothymic and anxious affective temperaments are characteristic to Crohn's disease, while UC patients did not differ in regard to these temperaments from the control group. Those findings accentuate the differences of the temperament profile in both diseases and therefore support the potential theory that the pathogenesis of mood disorders in both IBDs may have separate basis.

Depression is a heterogeneous condition triggered by various etiological factors, including genetic (neurotransmitter polymorphisms) and environmental ones (e.g. stress, immune infections). It has been suggested that chronic stress may cause alterations in synaptic transmission or change the morphology of neuron's structure–i.e. impair neuroplasticity processes—within particular brain regions, resulting in the development of depressive symptoms. Diminished 5-HT neurotransmission might attenuate hippocampal neurogenesis, and thus, contribute to the occurrence of depressive symptoms [41]. In IBD, which is a condition characterized by chronic inflammation, stress-related factors might affect neuroplasticity in central nervous system and hence predispose to mood disorders. In their study Hong et al. observed significant differences in CT scans between UC and healthy controls. Authors suggest that the reorganization of grey matter in IBD may stem from chronic gut inflammation processes [42]. However, more research needs to be done in regard to this field.

Depressive temperament exhibits low energy, non-assertiveness, negative cognition or being shy, highly pessimistic and gloomy [20,43]. Individuals with higher cyclothymic temperament have tendencies to rapid mood shifts between the depressive and hyperthymic traits—between high and low moods [44]. They are instable considering their energy, self-esteem, as well as activity in social life [45]. Patients with high scores on anxious temperament displays behavioral and emotional response to the potential threats in the environment, i.e. the tendency to excessive worrying and inability to relax [22].

Hypertymic temperament seems to be independent of other temperaments. It is suggested that affective temperaments are grouped into “cyclothymic-sensitive” with dysthymic, cyclothymic, irritable, anxious temperaments and into independent hypertimic temperament. In this way, a different role of hypertimic temperament is explained in the process of modifying the clinical course of various diseases [46].

Many studies have assessed the relation between affective temperament and the susceptibility to depressive and anxiety disorders [19,43–45].

Table 5 presents the correlation between affective temperaments and both HADS-A and HADS-D scales in CD and UC individuals. Our findings are consistent with the literature describing the association between affective temperaments (depressive, anxious, cyclothymic and irritable) with the high prevalence of anxiety and depression [44,48]. Results from the study of Kesebir et al. indicate that higher scores of depressive, anxious, cyclothymic and irritable dimensions are significantly higher in depressive individuals than in healthy ones [47]. Research evaluating ankylosing spondylitis (AS) yield following results: depressive, cyclothymic, irritable and anxious temperament scores correlated with Beck Anxiety Inventory and Beck Depressive Inventory, hence greater scores of depressive and anxiety scales [25]. Based on the abovementioned literature, we assume that the greater scores of depressive, cyclothymic, irritable and anxious temperaments in TEMPS-A the higher vulnerability to falling for mood disorders in IBD patients. Our group of patients did not qualified as depressed or anxious according to the results of HADS scales, however we suggest that the regular monitoring of CD patients is needed due to their TEMPS-A results, and following proneness to developing mood disorders.

Interestingly, UC patients showed significantly lower scores in irritable temperament in comparison to CD and control group. Irritable temperament is portrayed as an unstable mixture of dysthymic and hyperthymic features. This suggests that UC patients may be less dysphoric and aggressive, show less criticism, are less complaining in comparison to control and CD groups [45]. Irritable temperament has been reported to be a predictor of depression in many studies, also in the group of patients suffering from a polycystic ovary syndrome [49]. According to our data, UC patients are less likely to develop depressive symptoms on the basis of their core personality traits showed by TEMPS-A.

Hyperthymic temperament displays exuberant, high self-esteem, narcissism and higher sociability [50]. In our results hyperthymic temperament negatively correlated with both HADS scales. Those results are consistent with the literature describing the potential protective effect of hyperthymic temperament on mood disorders [51].

Literature presents many correlations between affective temperament and autoimmune diseases such as psoriasis, AS or rheumathoid arthritis [25,26,52]. The study evaluating patients with psoriasis did not show any significant temperament differences with control group. However, depressive and anxious temperaments were associated with stressful factor of the disease in a group of women [26]. Also in the study assessing TEMPS-A scores in the group of AS patients, researchers did not observe any differences in temperament between AS and control group [25]. However, results showed positive correlation between depressive, cyclothymic, anxious temperament and AS activity index. Described temperaments were also associated with greater pain, measured in visual analogue scale (VAS), in contrast to hyperthymic dimension which showed negative correlation to VAS. Affective temperament is associated with disease activity and pain in AS patients and could be a risk factor for depression and anxiety in this group of patients [25].

These examples are consistent with results of our study displayed in Tables 4 and 7. Depressive, cyclothymic and anxious temperaments showed positive correlation with scores of subjective aspects of Harvey-Bradshaw Scale, Crohn Disease Activity Index and Mayo Self Report utilized in assessing the activity of CD and UC. Interestingly, we did not observe any correlation between affective temperament and objective scales. Affective temperament significantly affects stress-related processes like in autoimmune diseases [53]. Individuals who present more vulnerable temperament traits, according to TEMPS-A scores, may acquire greater negative consequences of the stressor than those with more protective temperament [54]. We assume, that in this regard temperament can be identified as a specific risk factor to the development of mood disorders. Our findings imply that the course of the disease may be more harmful for patients possessing such personality traits and they may be more vulnerable in developing mood disorders. Additionally, in all scales hyperthymic temperament showed the negative correlation proving it's protective effects.

Affective temperament is perceived as being heritable [55]. A meta-analysis of CD and UC genome-wide association scans observed that IBD loci overlapped with immune-mediated diseases; the largest pertained to AS and psoriasis [56]. Therefore, it is very interesting that we have received similar temperament results like in studies which evaluated affective temperament in both diseases. It may suggest that those disorders share common genes encoding temperament traits, resulting in greater risk of psychiatric comorbidities. We have observed the significant temperament differences between CD and UC. Hence, we suggest that more genetic studies are needed to extract more data about common loci of UC and other immune-related diseases. Presumably, only CD shares the same genes responsible for affective temperament unlike UC.

The intestinal serotonergic signaling system plays an important and multifactorial role in IBD, e.g. affects inflammatory processes and the intensity of symptoms [57]. Gonda et al. showed the association between affective temperaments and S-allele of 5-HTTLPR polymorphism of the serotonin transporter (SERT) gene [58]. Hyperthymic component was the only one which did not show such association. S-allele of 5-HTTLPR is considered to be related with higher predisposition to depression [59]. Table 6 shows the positive correlation between both cyclothymic and anxious temperaments, and the number of soft stools, however hyperthymic temperament showed negative correlation in this aspect. Such results indicate the overt connection between affective temperament and the serotoninergic system in CD. Unfortunately, the association between SERT expression in IBD still requires more data, due to scarce literature. More comprehensive investigations are needed, especially those focusing on CD and UC separately, not collectively. Especially, that results of TEMPS-A imply significant differences in the serotoninergic transmission in those two disorders.

## Conclusions

In conclusions, to our knowledge, this is the first study evaluating the affective temperament via TEMPS-A in both CD and UC patients. The results of our study indicate significant differences between CD and UC due to temperament traits, and suggest distinct pathogenesis of mood disorders in IBD. Our findings show strong association between cyclothymic, depressive and anxious temperament and CD group in comparison to control groups. Also these temperaments positively correlated with subjective scales assessing the activity of UC and CD, and the number of loose stools in regard to CD scales. We have observed negative correlation of hyperthymic temperament in this regard, showing it's putatively protective effect on the disease course. We suggest that the mechanisms responsible for such findings are related to genetic polymorphisms and changes in serotoninergic neurotransmission, however more studies are needed to prove this association. TEMPS-A constitutes the simple tool, which could be utilized in the assessment of the vulnerability to psychiatric disorders and prognostic evaluation in IBD patients. Our results also indicate that analysis of affective temperament may contribute to genetic studies in identifying genes responsible for the development of affective disorders in IBD population. Creating more homogenous groups of patients regarding clinical manifestation and genetic profile, might results in administration of individualized and better adjusted treatment strategies against mood disorders in both CD and UC patients.

### Limitations

Main limitations of our study are: the cross-sectional type of the study, which does not allow the generalization of the findings and relatively small sample of patients for better assessment of the affective temperament. We also did not collect the data about participants who declined participating in the study or who were excluded from the research including their demographic, clinical or psychological characteristics. We were also unable to display exact mechanisms which could explain our findings, due to very complex network of dependencies.

## Supporting information

S1 File gastro TEMPS_3 anonim_Eng.xlsx. Data base of affective temperament in IBD patients.
(XLSX)

S2 File gastro baza_12 anonim_ENG.xlsx. Data base of clinical factors and surgical treatment of IBD patients.
(XLSX)
